# Controlled expression of avian pre-migratory fattening influences indices of innate immunity

**DOI:** 10.1242/bio.060018

**Published:** 2024-01-22

**Authors:** Marcin Tobolka, Zuzanna Zielińska, Leonida Fusani, Nikolaus Huber, Ivan Maggini, Gianni Pola, Valeria Marasco

**Affiliations:** ^1^ Department of Zoology, Poznań University of Life Sciences, Wojska Polskiego 71c, 60-625 Poznan, Poland; ^2^ Department of Interdisciplinary Life Sciences, Konrad Lorenz Institute of Ethology, University of Veterinary Medicine Vienna, Savoyenstraße 1a, 1160 Vienna, Austria; ^3^ Department of Behavioural and Cognitive Biology, University Biology Building, University of Vienna, Djerassiplatz 1, 1030 Vienna, Austria; ^4^ Department for Farm Animals and Veterinary Public Health, University of Veterinary Medicine Vienna, Veterinärplatz 1, 1210 Vienna, Austria; ^5^ Istituto Sperimentale Zootecnico per la Sicilia, via Roccazzo 85, 90135 Palermo, Italia; ^6^ Department of Interdisciplinary Life Sciences, Research Institute for Wildlife and Ecology, University of Veterinary Medicine Vienna, Savoyenstraße 1a, 1160 Vienna, Austria

**Keywords:** Leukocyte coping capacity, Hemagglutination, Hemolysis, Innate immunity, Migratory fattening, Avian migration

## Abstract

While immunity is frequently dampened when birds engage in strenuous migratory flights, whether and how immunity changes during the rapid accumulation of energy stores in preparation for migration remains largely unknown. Here we induced pre-migratory fattening through controlled changes of daylight in common quails (*Coturnix coturnix*) and regularly assessed changes in three markers of constitutive innate immunity (leukocyte coping capacity or LCC, hemagglutination and hemolysis titres) and measures of body composition (lean and fat mass). All the three markers showed similar changes over the pre-migratory fattening process. LCC responses, hemagglutination titres, and hemolysis titres, were on average higher in the mid-fattening phase compared to the peak-fattening phase, when values were similar to those observed prior the start of pre-migratory fattening. At mid-fattening, we found that the birds that showed a larger accumulation of fat mass (as % of body mass) had lower LCC peak responses and hemolysis titres. Reversibly, at mid-fattening, we also found that the birds that kept a higher proportion of lean mass (as % of body mass) had the highest LCC peaks. Our results indicate that migratory birds undergo changes in immune indices (over 8 weeks) as they accumulate energy stores for migration and propose that this could be due to competing or trade-off processes between metabolic remodelling and innate immune system function.

## Teaser

**Summary:** Immunity is vital when migrating to new environments. It is costly and competitive to other physiological processes. Here, we bring new evidence on this process in migratory birds.

## INTRODUCTION

Birds show various physiological adaptations to accommodate the intense energy demands of long-distance migratory flights (Cornelius et al., 2013). One of the most spectacular preparatory features described so far is pre-migratory fattening – a rapid accumulation of energy stores prior to the onset of active migration (Bairlein, 2002; Jenni and Jenni-Eiermann, 1998; Jenni and Schaub, 2003). Studies across different bird species sampled at stopover sites (i.e. where migrating birds temporarily suspend the long-distance endurance flight to rest and refuel) show that individuals with larger fat stores can migrate faster (Brust et al., 2022; Sjöberg et al., 2015). Thus, the physiological and metabolic preparations (e.g. hyperphagia, fattening, and flight muscle hypertrophy) are vital for successful migration (Bairlein, 2002; Guglielmo, 2018; Hedenström and Alerstam, 1997).

Due to their mobility, migratory animals are exposed to a higher number and greater variety of pathogens compared to non-migratory populations (Buehler et al., 2010; van Dijk et al., 2014). Thus, physiological adaptations associated with the maintenance of immune system functioning are particularly relevant to minimise the negative effects of infectious diseases in migratory species (Eikenaar et al., 2023; Fritzsche McKay and Hoye, 2016; Roitt et al., 2001). However, maintaining a high immune response might require adjustments in the amount of resources allocated to the system relative to other competing behavioural and physiological processes (Germain, 2012; Norris and Evans, 2000; Sheldon and Verhulst, 1996). For example, nutritional deficiencies often alter the development of the immune system and impair its optimal function (Hotamisligil, 2006; Lochmiller et al., 1993; Mauck et al., 2005; Samartín and Chandra, 2001). Adequate fine-tuning of effective immune responses is likely to be even more difficult in migratory species because they have to rapidly adjust their energy requirements in preparation for migration (pre-migratory fuelling), and between repeated stopovers and strenuous migratory flights (Altizer et al., 2011; Buehler et al., 2010; Eikenaar et al., 2023; Srygley and Lorch, 2011; Voigt et al., 2020).

Several studies in birds suggested that migrants temporarily suppress immunity and re-allocate energy to endurance flights (Altizer et al., 2011; Eikenaar and Hegemann, 2016; Eikenaar et al., 2018; Møller et al., 2004; Nebel et al., 2012, 2013; Owen and Moore, 2006, 2008a,b). However, it is still unclear whether and how immunity might be optimally modulated during pre-migratory fuelling and to what extent individual variation of innate immunity is related to the rapid accumulation of energy stores during this specific life cycle stage. Recent studies in the northern wheatear (*Oenanthe oenanthe*) showed that energy stores of free-living birds sampled at a stopover site during autumn migration were positively correlated with immune parameters, and *ad libitum* feeding of temporarily caged individuals led to rapid increases in their blood bacterial killing capacity (Eikenaar et al., 2020a,b). It could be hypothesised that the accumulation of energy stores is associated with a potentiation of immune responses. On the other hand, immunological variation during pre-migratory fuelling might also be the consequence of a potential competition between the energy required for fat accumulation and/or muscle volume increases and the need to maintain a ‘competent’ immune system. Here, we explored these two possibilities by experimentally controlling the migratory state of captive young adult common quails (*Coturnix coturnix*, Linnaeus, 1758) to simulate autumn pre-migratory fattening. We assessed potential changes in leukocyte oxidative burst capacity, referred to as leukocyte coping capacity (hereafter LCC). LCC is a functional measure of early innate immune protection in response to a secondary external stimulus (McLaren et al., 2003). Additionally, we evaluated natural antibodies (NABs) and complement capacity through hemagglutination and hemolysis titres, hereafter referred to as agglutination and lysis. These constitutive innate immune indices assess the agglutination or lytic capacity of NABs in bird plasma towards mammal red blood cells (Matson et al., 2005). We measured the three described parameters during three different physiological states: (1) before pre-migratory fattening, (2) at mid-pre-migratory fattening, and (3) at the pre-migratory fattening peak. Further, we assessed whether shifts in the selected innate immune markers were associated to within-individual changes in body composition (lean and fat mass) over these three phases.

## RESULTS

### Effects of photoperiod manipulation on pre-migratory fattening

**Fig. 1.**
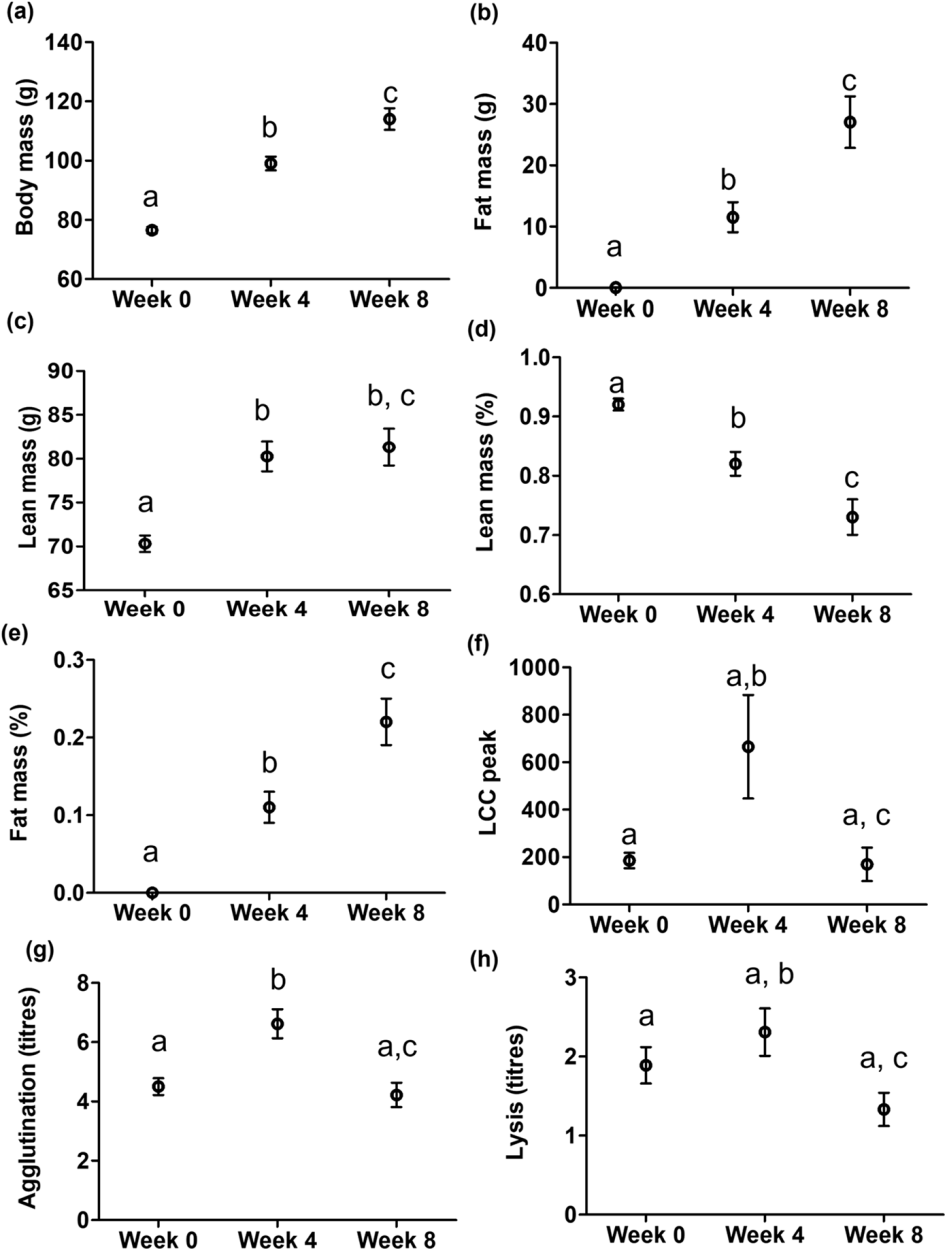
**Changes in (A) body mass (g), (B) fat mass (g), (C) lean mass (g), (D) lean mass as % of body mass, (E) fat mass as % of body mass, (F) LCC peak levels, (G) hemagglutination titres, and (H) hemolysis titres at each photoperiod sampling time point, before the start of pre-migratory fattening (week 0), at mid-pre-migratory fattening (week 4), and at the pre-migratory fattening peak (week 8) in a population of common quails.** Sample sizes in A-E, G, H: week 0, *n*=28; week 4, *n*=16, and week 8, *n*=18; in F: week 0, *n*=18, week 4, *n*=15, and week 8, *n*=16. Data are indicated as means±se, different letters indicate pairwise statistical differences between the differing photoperiod sampling time point (*P*<0.05); in (F) week 0 versus week 4, *P=*0.05, week 0 versus week 8, *P=*0.06.

Body and fat mass increased linearly in relation to shortening day length in both male and female quails (Table 1A,B, post-hoc contrasts: *P*≤0.003 for all; Fig. 1A,B). Lean mass increased during the first phase of pre-migratory fattening (week 0-week 4; post-hoc *P<*0.001), but remained relatively stable in the following 4 weeks, week 4-week 8 (Table 1C, post-hoc: *P=*0.7; Fig. 1C). The proportion of lean mass to body mass linearly decreased over time (Table 1D, post-hoc contrasts: *P*≤0.004 for all; Fig. 1D) and the proportion of fat mass to body mass showed the opposite pattern (Table 1E, post-hoc contrasts: *P*≤0.001 for all; Fig. 1E).

**Table 1.**
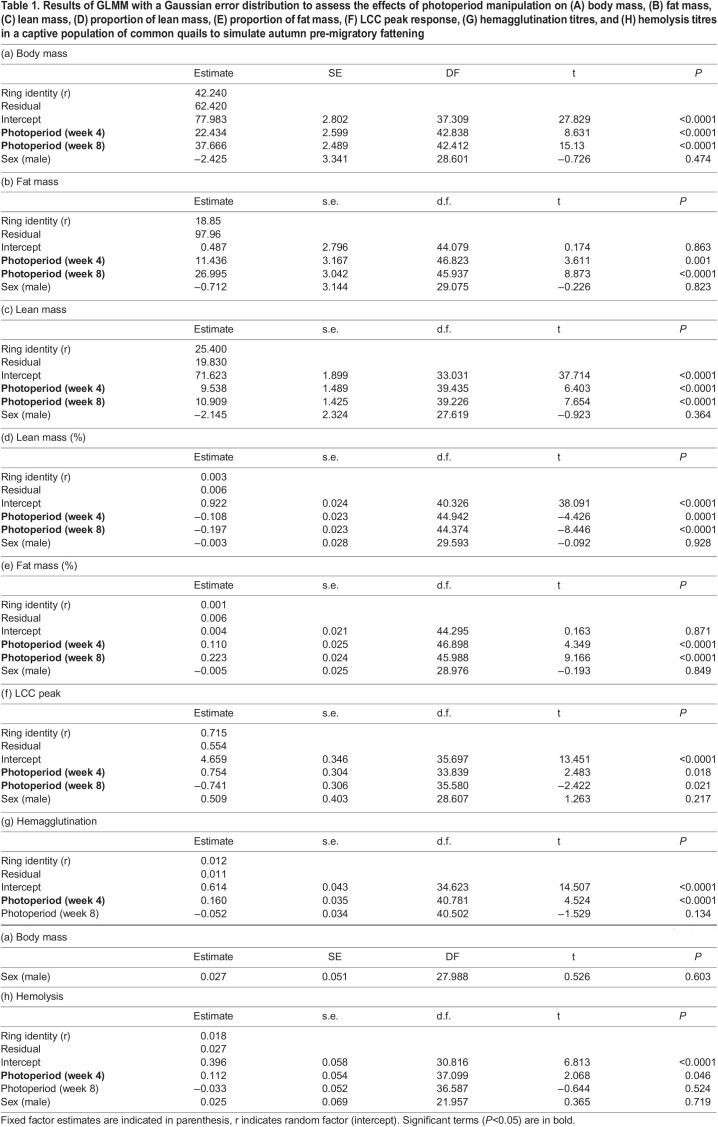
Antibodies for western blotting and immunofluorescence

### Effects of photoperiod manipulation on LCC peak levels, agglutination and lysis titres

LCC peak levels differed among sampling weeks (Table 1F). LCC peak values tended to be on average higher at week 4 compared to week 0 (*P=*0.05, Fig. 1F); at week 8, LCC peak levels were lower compared to week 4 (*P=*0.0001, Fig. 1F), but not substantially lower compared to week 0 (*P=*0.06, Fig. 1F). There was no effect of sex on LCC peak levels (Table 1F). Similarly, a bell-shaped pattern was observed for the agglutination titres as days became shorter (Fig. 1G). Agglutination titres were, on average, higher at week 4 compared to week 0 (*P=*0.0002, Fig. 1G), as well as to week 8 (*P<*0.0001, Fig. 1G) when values reached similar levels as at week 0 (*P=*0.3, Fig. 1G). We found no effects of sex on agglutination (Table 1G). Lysis titres showed the largest values on average at experimental week 4 (Table 1H), though significant differences were only detected between week 4 and week 8 (*P=*0.04; *P*≥0.1 for the other two pairwise contrasts, Fig. 1H). We found no effect of sex on lysis titres (Table 1H).

For all three markers, results did not change qualitatively when using only the subset of birds that were repeatedly sampled across the three sampling time points (Table S2, Supplementary Material).

### Relationships between energy stores, LCC peak, hemagglutination and hemolysis titres

**Fig. 2.**
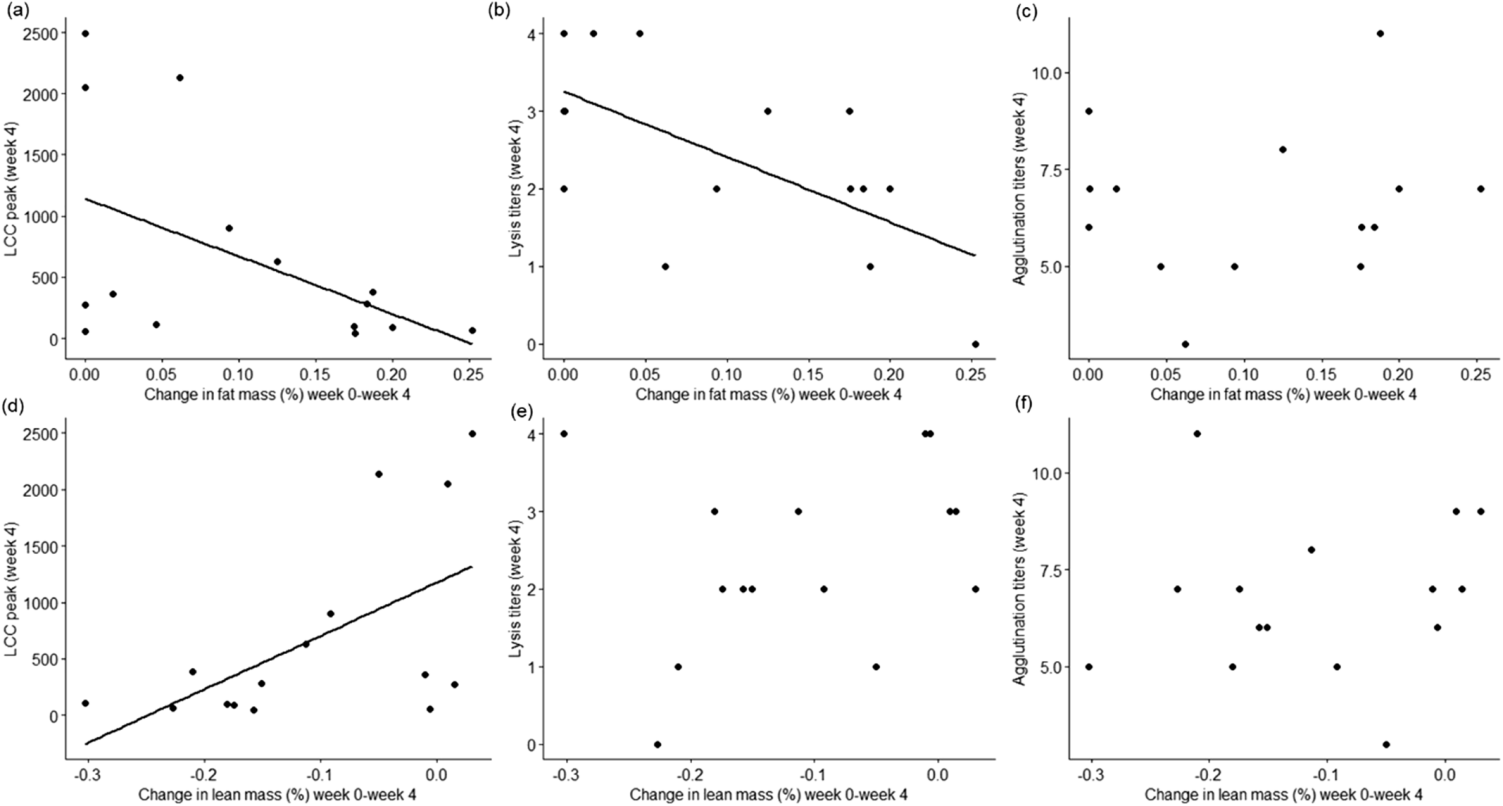
**Correlation plots between changes in fat mass as proportion of body mass (A-C) or changes in lean mass as proportion of body mass (D-F) between week 0 and week 4 and each immune maker measured at week 4 (i.e. LCC peak responses, hemagglutination or hemolysis titres).** In all panels, dots represent individual samples, regression lines are shown only for significant correlations (see Results).

The proportional increase in fat mass (as % of body mass) between week 0 and week 4 was negatively associated with LCC peak levels found at week 4 (estimate±SE: −0.06±0.01, t=−4.40, *P=*0.001; Fig. 2A) and with lysis titres at week 4 (estimate±s.e.: −0.07±0.01, t=−5.20, *P=*0.0003, Fig. 2B), while this variable was not linked with agglutination titres at week 4 (*P=*0.92, Fig. 2C, full statistics in Table S3, Supplementary Material). Reversibly, the change in lean mass (as % of body mass) was positively related to LCC peak responses measured at week 4 (estimate±s.e.: 0.07±0.02, t=2.86, *P=*0.02, Fig. 2D), but not with the other two markers (*P*≥0.1, Fig. 2E,F and Table S3, Supplementary Material). We found no link between either the change in lean mass or fat mass (as % of body mass) observed between week 4 and week 8 with any of the immune markers measured at week 8 (*P*≥0.1, Table S3 and Fig. S3, Supplementary Material).

## DISCUSSION

In this study we controlled the expression of autumn pre-migratory fattening in age-matched adult common quails and showed that LCC peak responses, agglutination and lysis titres varied markedly along the pre-migratory fattening process. We found that LCC peak responses, agglutination titres, and to some extent lysis titres were on average higher in the mid-fattening phase (week 4), but by the time quails achieved their peak fat load (week 8), all three markers decreased to levels comparable to those prior the start of pre-migratory fattening (week 0). These non-linear changes in all three innate immune markers were surprising and provide limited support for the general hypothesis that birds, as other migrating organisms, downregulate immune system functioning before migration (Altizer et al., 2011; Eikenaar and Hegemann, 2016; Owen and Moore, 2006; Rogers et al., 2022).

The increase in LCC peak levels, agglutination and, to some degree, lysis titres, which we found 4 weeks after the beginning of pre-migratory fattening was not expected. It suggests that the immune system undergoes substantial remodelling during pre-migratory fattening as birds prepare for long-distance flights. It is worth highlighting that all three immunological markers changed in a similar pattern, suggesting they all reflect innate immune capacity. Variation in immune responses over larger spans of the life cycle has been previously reported in birds. For example, a study in wild skylarks (*Alauda arvensis*) showed that some components of innate immune defence (lysis and agglutination) were highest during pre-migratory moulting, while concentrations of monocytes and basophils increased during autumn migration, and eosinophil concentration was highest during spring migration (Hegemann et al., 2012). However, the measurements of immune markers from the latter work were not the same across the study years and the birds' age could not be estimated, which adds a further level of complexity for data interpretation (Buehler et al., 2009). On the other hand, a study of red knot (*Calidris canutus*) under controlled conditions using birds of similar age clearly showed changes in immune strategies across different stages of the annual cycle, suggesting increased immunological costs during the period of body mass change (fattening and spring migration) compared to the periods when body mass was stable (Buehler et al., 2008). We propose that examinations over a shorter time scale like in the present study can be extremely useful to understand a potential link with behavioural and metabolic strategies characterising the distinct stages of the migratory period.

The Echo-MRI data confirmed that pre-migratory fuelling was mainly related to intense accumulation of fat stores and less to the increase of lean mass, which would at least in part reflect increases in pectoral flight muscles (Boswell et al., 1993; Guglielmo, 2018; Price et al., 2011). We did find a significant increase in lean mass, but this effect was limited to the first 4 weeks of the pre-migratory fattening process. Furthermore, we only found a relationship between LCC peak, lysis titres, and changes in body composition at week 4, when the birds showing the highest LCC peaks at week 4 kept a higher proportion of lean mass (as % of body mass), and, therefore, also accumulated smaller fat stores. Similarly, the birds showing the highest lysis scores were those that accumulated smaller fat stores, suggesting that these two innate immune markers undergo similar functional changes as birds prepare for migration. However, there was no evident link between lysis scores and changes in lean mass, nor were agglutionation titres associated with changes in fat or lean masses. This result might be due to strategic shifts between metabolic remodelling (catabolic/anabolic) and innate immune capacities (Norris and Evans, 2000; Sheldon and Verhulst, 1996). Alternatively, birds that showed the highest LCC peaks or lysis scores may have delayed the accumulation of fat stores due to physiological constraints possibly associated to oxidative stress or resource allocation. A previous study in quails showed that the experimental control of the migratory state is associated with pronounced tissue-specific changes in oxidative status, with pectoral muscle antioxidants (glutathione peroxidase) being (a) positively related to increases in subcutaneous fat stores as the birds transitioned to a migratory state, and (b) negatively related to losses in fat stores as the birds transitioned back to a non-migratory state (Marasco et al., 2021).

Activated leukocytes produce free oxygen radicals in the frame of the so-called oxidative burst (McLaren et al., 2003) and reactive oxygen species (ROS) play a central role in the orchestration of innate and adaptive immunity (Bogdan et al., 2000; Kohchi et al., 2009). Thus, decreasing LCC as well as overall number of leukocytes, may be an important component of oxidative balance regulation, protecting organisms from oxidative damage and inflammation during energy challenges such as migration (Cywińska et al., 2010; Dick and Guglielmo, 2019; Nieman, 2000; Voigt et al., 2020). Lysis assays indicate the activity of complement proteins, crucial in the immune system for promoting the lysis of non-self cells and viruses. This assay evaluates the complement system's ability to lyse red blood cells, and, when considered alongside other measures, such as LCC, it can be interpreted as an assessment of overall early innate immune competence (Ochsenbein et al., 1999). Consequently, the lowest scores for lysis we found in the birds with the highest increases in fat masses may suggest a trade-off between energy accumulation and the production of complement proteins. On the other hand, agglutination assays assess the capacity of antibodies to recognise and bind to foreign antigens, such as those present on the surface of red blood cells (Matson et al., 2006). The lack of correlation with body composition measures is surprising, given that hemagglutination is considered a component in the cascade of the same innate immune response (Matson et al., 2005).

Our experimental design does not allow us to clearly discriminate between the actual effects of the altered photoperiod regime and any other environmental or biological factors that may have contributed to the observed changes in the measured innate immune markers. We feel that we can rule out the former as the experiment was performed in controlled/constant ambient temperature conditions and in well standardised housing and sampling procedures. A potential biological contributory factor to the effects of the altered daylight regime could be associated with age-related changes in immune functioning, which do occur in relation to ageing processes (Shaw et al., 2013). However, we can also safely exclude that this would be the case in our experiment as the birds were all young adults when the experiment started (6 weeks old) and the entire procedures lasted over a 8-week period, thus well before the start of organismal ageing in quail species, which is expected to emerge after 2 years of age (Ottinger et al., 2004). It is also improbable that the observed changes in immune functions are a result of the maturation process, as we would anticipate a continuous increase (Aastrup and Hegemann, 2021) rather than the bell-shaped pattern we found in our study. In addition, the similar dynamics of LCC and lysis linked with changes in lean and fat mass over the mid pre-migratory fattening period strongly suggest that the changes in innate immune function we found are most likely attributable to pre-migratory fuelling remodelling rather than to changes related to the increasing age of the experimental birds.

To summarise, our results suggest that the process of pre-migratory fattening is associated with similar dynamic changes in LCC peak responses, agglutination and lysis titres. These three immune indices are integral components of the innate immune system, which acts rapidly and effectively against a broad range of pathogens, even without prior exposure. This aspect is crucial in the context of animal migration, where the ability to respond quickly to various threats is central for fitness. In future work, experimental manipulations involving changes in resources (for instance, limited food availability) during pre-migratory fuelling would be needed to test the existence of physiological trade-offs with immune function. We also point out that studies using multiple markers/indices of both innate and acquired immunity would be necessary to obtain detailed information on seasonal trade-offs and remodelling of the immune system (distinguishing between pre-migrants and actively migrating birds), and its function in protecting migrants from disease and infectious risk during the most energetically demanding phases of their life cycle.

## MATERIALS AND METHODS

### Ethical statement

The experiment was performed in compliance with Austrian legislation and the approval of the Ethics Committee of the University of Veterinary Medicine Vienna and the Federal Ministry of Science, Research and Economy (2021-0.466.199).

### Study subjects and photoperiod manipulation

Fertile eggs of common quails were obtained from a breeding stock population kept at the Istituto Sperimentale Zootecnico per la Sicilia (ISZS, Palermo, Italy), which originated from wild common quail founders (Smith et al., 2018). After transportation to the Konrad Lorenz Institute of Ethology (Vetmeduni, Vienna), the eggs were artificially incubated and hatchlings reared under a 16:8 h light:dark cycle until 4 weeks of age when they were sexed and moved to enclosures of 80×100×210 cm in sex-mixed groups of 11-12 birds (*n*=3) until the termination of the experiment (28 birds in total). Food (turkey starter, Lagerhaus, Austria) and water were always provided *ad libitum*. All birds were maintained in photoperiod- and climate-controlled indoor animal facility rooms at 20-24°C. When the birds were 6 weeks of age (i.e. week 0 of pre-migration fattening), they were exposed to a gradual reduction of day length (30 min/week) over eight consecutive weeks until the photoperiod reached 12:12 h light:dark. This light:dark schedule simulates autumn migration and triggers pre-migratory fattening (Marasco et al., 2021).

### Measurements of innate immune responses: whole blood LCC, hemagglutination and hemolysis titres

Birds were sampled at week 0 at 16:8 h light:dark, at week 4 at 14:10 h light:dark, and at week 8 at 12:12 h light:dark. Due to logistic reasons, there are differences in the number of within-individual repeated measures among the three sampling time points and across markers (full details in Table S1, Supplementary Material). At week 0, we took a blood sample for measurements of agglutination and lysis titres from all birds (11 females and 17 males, *n*=28); for practical/logistical reasons LCC measurements were performed in a subset of birds (8 females and 10 males, *n*=18). In the following sampling time points (week 4 and week 8), we excluded 10 birds from laboratory analyses (samples to be used for other purposes - to be published elsewhere). Thus, sample sizes at week 4 and at week 8 were 16 birds (6 females and 10 males) and 18 birds (7 females and 11 males), respectively. At week 4, two birds showed signs of pecking and were not sampled. These were resolved by brief social isolation and the two individuals could be included again in the sampling at week 8. One day before blood sampling, the birds housed within the same enclosures were transferred into single cages (71×109×79 cm), but all birds remained in visual and acoustic contact with their conspecifics to minimise possible effects of the changes in the social environment. All birds were sampled within 3 min after opening the cage. As appropriate, one aliquot of collected blood was immediately used to perform LCC measurements; the remaining sample was spun (2000 rpm for 10 min) within 2 h of collection to separate plasma from red blood cells and stored at −80°C until later measurements of agglutination and lysis titres.

### LCC measurements

To measure unstimulated blood chemiluminescence levels, which provides information about the individual baseline level of reactive oxygen species (ROS), 10 μl of lithium heparinised whole blood were transferred into a silicon anti-reflective tube (Lumivial, EG & G Berthold, Germany). We added 90 μl of 0.25×10^−3^ M Lucigenin (bis-N-methylacridinium nitrate; Sigma-Aldrich) dissolved in dimethyl sulfoxide (DMSO; Merck, Darmstadt, Germany) and diluted with phosphate-buffered saline (PBS, pH 7.4). Subsequently, 10 μl of PBS were added and the tube was gently swirled for mixing. Lucigenin produces chemiluminescence when combined with an oxidant (i.e. superoxide anion), resulting in a low-intensity light reaction (Li et al., 1998). To measure full blood chemiluminescence produced in response to a secondary challenge, we prepared a second tube in parallel as described above but added 10 μl of 10^−5^ M phorbol 12-myristate 13-acetate (PMA; Merck, Darmstadt, Germany) instead of 10 μl PBS. Blood chemiluminescence for each tube was assessed every 10 min for 30 s over a period of 70 min and expressed in relative light units using a portable high-sensitivity chemiluminometer (Junior LB 9509, EG & G Berthold, Germany). When not in the chemiluminometer, tubes were incubated at 38°C in a lightproof metal bead bath (Minitüb, Tiefenbach, Germany). To correct for background noise, we subtracted the values of the control sample from those of the challenged sample measured at the same time point. From the resulting LCC response curve, we extracted the LCC peak, which was defined as the maximum ROS production in the course of the induced oxidative burst. This variable was a reliable proxy of the entire LCC response over time (adj. R^2^=0.94, *P*<0.0001, see Figs S1,2, Supplementary Material), as shown in previous work in birds and mammals (Huber et al., 2019, 2020; Hansen et al., 2020).

### Hemagglutination and hemolysis titres

To measure NAB activity stimulated by antigens, we followed the protocol proposed by Matson et al. (2005). As stimuli, we used rabbit red blood cells washed five times with PBS and, based on the hematocrit measure, diluted to a 1% concentration. On a 96-well (eight rows by 12 columns), round (U) bottom assay plates, we pipetted thirteen microliters of quail plasma into columns 1 and 2 of the plate and 13 μl of 0.01 M PBS into columns 2-12. Using a multi-channel pipette, we serially diluted (1:2) through column 11 the contents of the column 2 wells, resulting in dilutions ranging from 1 to 1/1024 with 13 μl in every well. The 13 μl of PBS in column 12 acted as a negative control. Subsequently, we added 13 μl of a 1% rabbit blood cell suspension to all wells, effectively halving all plasma dilutions. Then we gently vortexed each plate for 10 s, which were sealed with parafilm, covered with a polystyrene plate lid, and incubated at 37°C for 90 min. Upon completion of the incubation, we tilted each plate to a 45° angle for 20 min at room temperature to enhance visualization of agglutination, and then scanned the plates. Afterwards, we kept plates at room temperature for an additional 70 min and scanned for a second time to record maximum lysis. From the digitised images, lysis and agglutination were scored for each sample. Both parameters were recorded as the negative log2 and scored from 0 to 10 according to observed activity in wells.

### Measurements of body mass and body composition using an Echo-MRI analyser

After collecting the blood sample, each bird was weighed to the nearest 0.01 g using an electronic balance. Subsequently, its lean and fat mass were measured to the nearest 0.01 g using a quantitative magnetic resonance analyser (Echo-MRI^TM^ Birds and Bats Body Composition Analyzer, Houston, USA) (Guglielmo et al., 2011). As at week 8 the birds were immediately euthanised for tissue analyses for separate experiments, the Echo-MRI measurements at week 8 were performed 2-4 days before the birds were blood sampled (the body mass of the birds on the day of the Echo-MRI scans and at the time of blood sampling were highly correlated: R=0.98, *P<*0.0001). The birds were placed into appropriately sized ventilated holding tubes (62 mm I.D.) inside the Echo-MRI analyser for approximately 3 min; each bird was scanned three times, and the average of the measurements was used for statistical analyses.

### Statistical analysis

Statistical analyses were performed in R v 3.6.2 (R Core Team, 2022) in RStudio v 1.3.1093 (R Studio Team, 2022) using general linear models (GLMs) or generalised linear mixed models (GLMMs) with a Gaussian distribution error [package “lme4” - (Bates et al., 2015)]. We assessed the effects of the photoperiod manipulation on proxies of migratory fattening (i.e. body mass, fat mass, and lean mass as well as fat mass and lean mass as a proportion of body mass), and immune indices (LCC peak levels, agglutination, and lysis titres) using separate GLMMs for each response variable. We entered photoperiod (week 0, week 4, and week 8), sex (male, female) and their interaction as fixed factors, while individual bird identity was entered as a random factor to control for the presence of repeated measurements. As the interaction of time and sex was always not significant (*P>*0.05) it was subsequently removed from the final models. LCC peak levels, agglutination and lysis titres were log-transformed to improve model residuals (we added a constant +1 to all lysis scores, which ranged 0-4). We excluded three LCC measurements from statistical analysis (one bird at week 4, two birds at week 8) as the control sample yielded extreme, unrealistic chemiluminescence levels. We used the R package ‘emmeans’ (Length et al., 2018) to perform pairwise post-hoc contrasts for significant outcomes in the main models (Tukey′s *P-*values adjustment). To assess the strength of within-individual effects, the GLMM model for the LCC, agglutination and lysis data were re-run using only the subset of birds that were repeatedly sampled across the three sampling photoperiod points (LCC: 8 birds; agglutination and lysis: 16 birds).

To further explore the effect of photoperiod on LCC peak levels, lysis, and agglutination titres found in these main models, we performed two separate GLMs to test whether the rate of fattening preceding the relevant blood sampling time point (i.e. calculated as the within-individual change in the proportion of fat, or lean mass in relation to body mass) was associated with the three immunological markers measured either at week 4 or at week 8; the markers were standardised in z-scores so that all three variables were made dimensionless. Multicollinearity among the immunological markers was always within acceptable range and low (variance inflation factor (VIF)<1.4, R package “car”). As the change in lean and fat mass (% of body mass) between week 0-week 4 and week 4-week 8 were highly correlated (-0.97<R<−0.74, *P*<0.0001), we tested each response variable (change in proportion of fat and change in proportion of lean mass in relation to body mass) separately.

## Supplementary Material

10.1242/biolopen.060018_sup1 Supplementary information
