# Supplementary material for: Controlled expression of avian pre-migratory fattening influences indices of innate immunity
Source: Biol Open. 2024 Jan 22;13(1):bio060018. doi: 10.1242/bio.060018 (PMC10836650; doi:10.1242/bio.060018)
Supplement: Supplementary information [file biolopen-13-060018-s1.pdf]

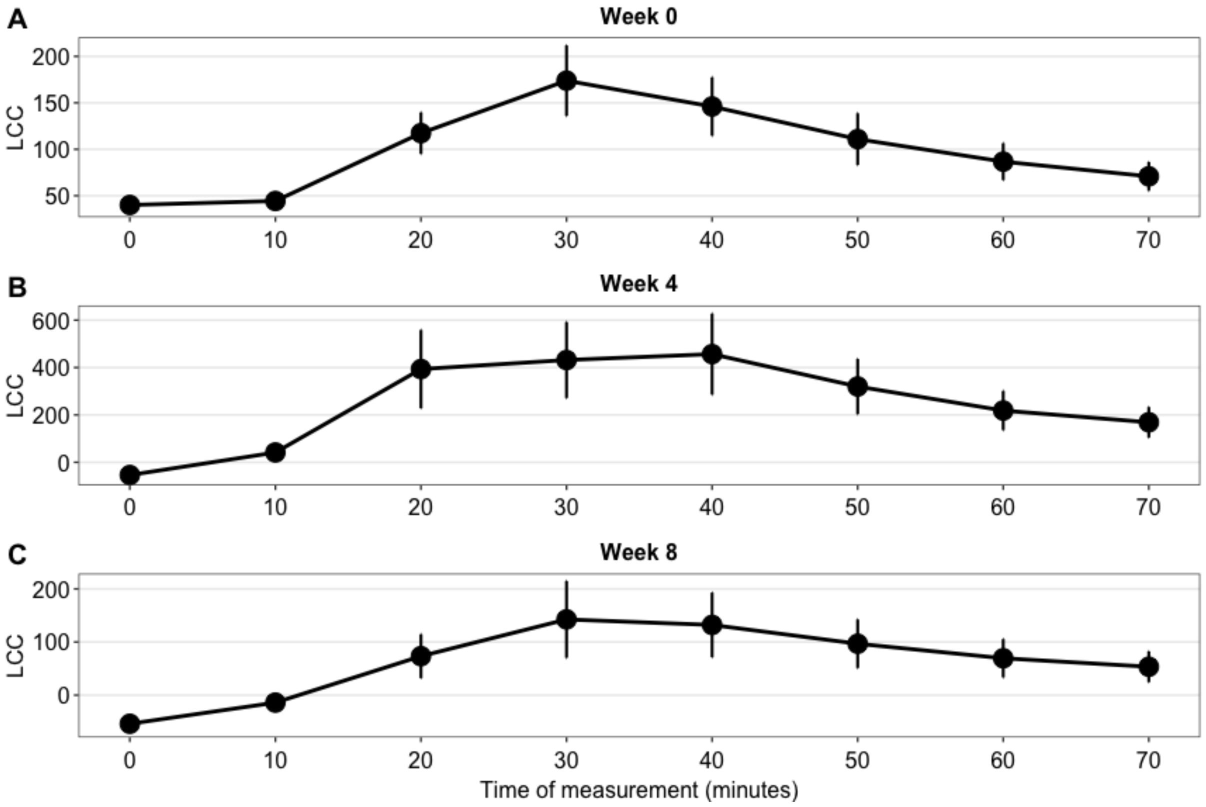

Fig. S1. Mean changes of the LCC values over the 70 minutes of measurements during the three sampling phases: (A) week 0, (B) week 4, and (C) week 8. Data are shown as mean ± se.

Biology Open • Supplementary information

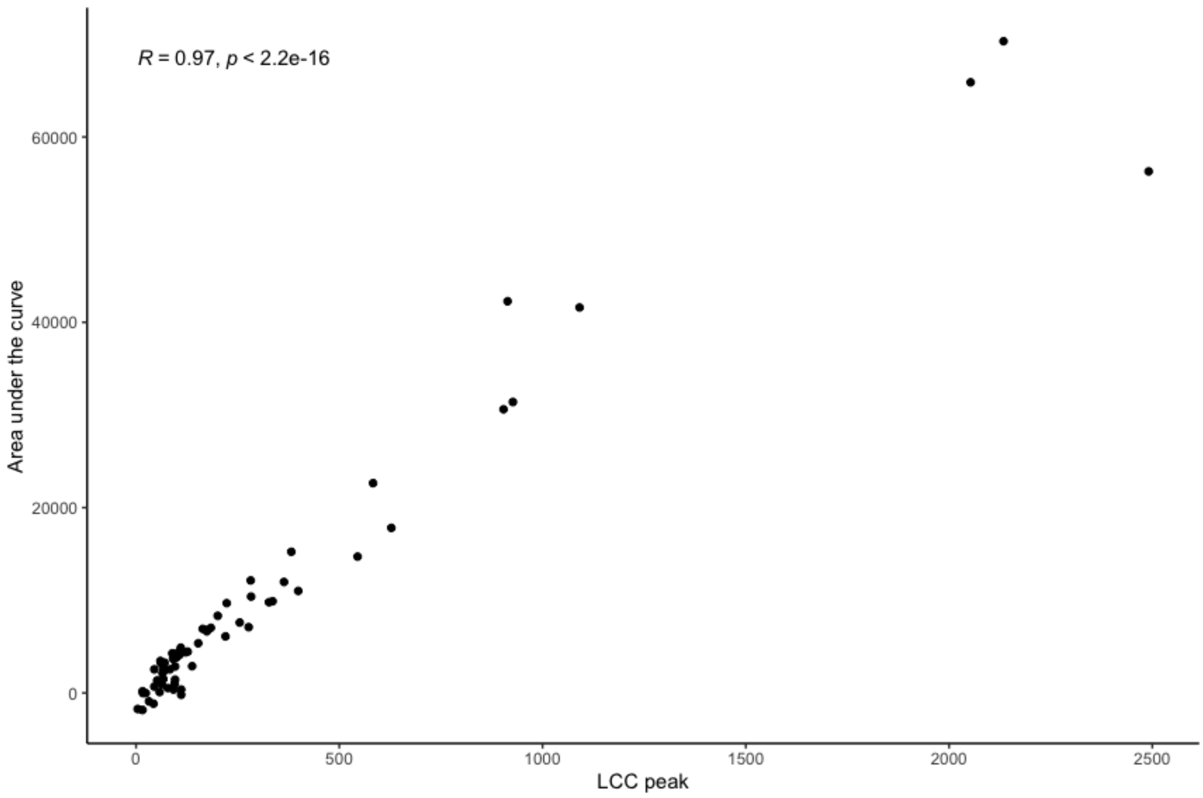

Fig. S2. Correlation between LCC peaks and areas under the curve over the 70 minutes of measurements during all three sampling phases. Dots indicate individual samples.

Biology Open • Supplementary information

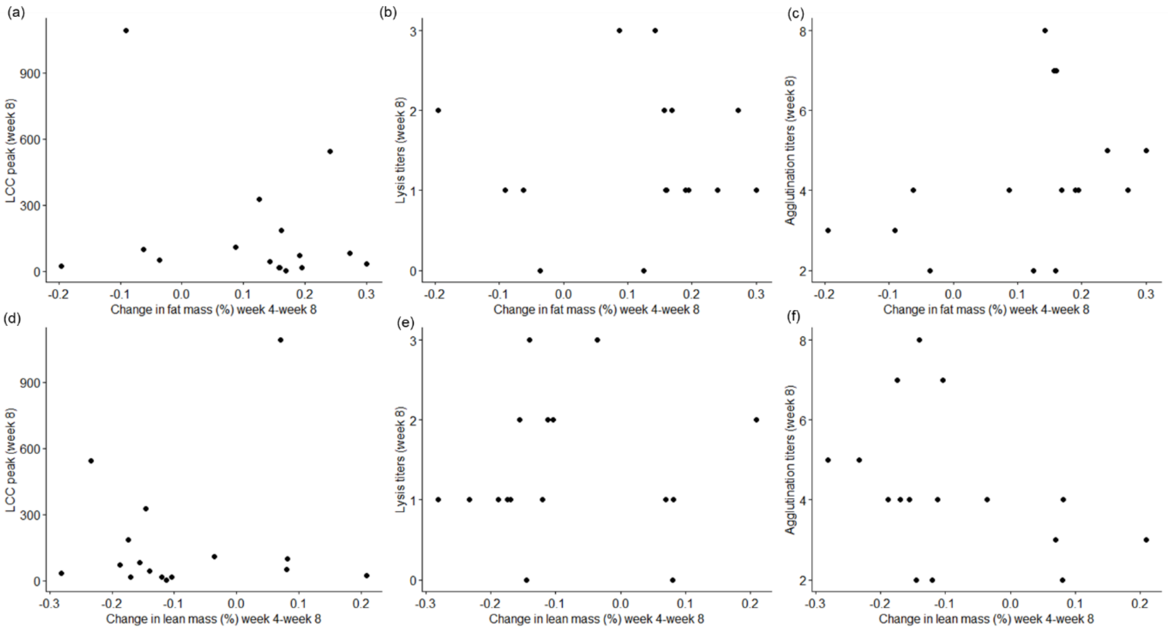

Fig. S3. Correlation plots between changes in fat mass as proportion of body mass (a-c) or changes in lean mass as proportion of body mass (d-f) between week 4 and week 8 and each immune maker measured at week 8 (i.e. LCC peak responses, lysis, or agglutination titres). In all panels, dots represent individual samples.

Table S1. Sampling regime across the three different photoperiod sampling time points (week 0, week 4, and week 8) for morphology assessment (body mass, fat and lean mass) and measurements of hemagglutination and hemolysis titres. In blue, the birds for which LCC measurements were additionally available. See Material and Methods in the main text for full detail on study design.

Week 0	Week 4	Week 8
343	343	343
344		344
345	345	345
347	347	347
349		
352		
353	353	353
354		
355	355	355
356	356	356
359	359	359
360	360	360
361		
362	362	362
365		365
366		
367		
368	368	368
370	370	370
371		
374	374	374
375	375	375
377	377	377
378		
379		
380	380	380
381		
383	383	383

Biology Open • Supplementary information

Table S2. Results of generalized linear mixed model with a Gaussian distribution error to assess the effects of photoperiod manipulation on (a) LCC peak response, (b) agglutionation, and (c) lysis titres in a captive population of common quails repeatedly sampled over week 0, week 4 and week 8 (LCC: n = 8 birds; lysis and agglutination: n = 16 birds). Fixed factor estimates are indicated in parenthesis, r indicates random factor (intercept). Significant terms (p < 0.05) are in bold.

(a) LCC

	**Estimate**	**SE**	**DF**	**t**	***p***
Ring identity (r)	1.230				
Residual	0.680				
Intercept	4.590	0.650	7.961	7.061	<0.0001
**Photoperiod (week 4)**	0.927	0.412	13.113	2.250	0.042
Photoperiod (week 8)	-0.606	0.433	13.262	-1.400	0.184
Sex (male)	0.446	0.858	6.105	0.520	0.621

Pairwise post-hoc contrasts: week 0 *vs* week 4, p = 0.1, week 4 *vs* week 8, p = 0.009, week 0 *vs* week 8, p = 0.4.

(b) Agglutination

	**Estimate**	**SE**	**DF**	**t**	***p***
Ring identity (r)	0.020				
Residual	0.010				
Intercept	0.624	0.062	18.145	10.015	<0.0001
**Photoperiod (week 4)**	0.148	0.038	30.000	3.862	0.001
Photoperiod (week 8)	-0.065	0.038	30.000	-1.702	0.099
Sex (male)	0.049	0.074	14.000	0.665	0.517

Pairwise post-hoc contrasts: week 0 vs week 4, p = 0.002, week 4 vs week 8, p < 0.0001, week 0 vs week 8, p = 0.2.

(c) Lysis

	**Estimate**	**SE**	**DF**	**t**	***p***
Ring identity (r)	0.020				
Residual	0.022				
Intercept	0.326	0.070	20.727	4.636	<0.0001
**Photoperiod (week 4)**	0.150	0.053	30.000	2.838	0.008
Photoperiod(week 8)	-0.011	0.053	30.000	-0.208	0.836
Sex (male)	0.018	0.080	14.000	0.229	0.822

Pairwise post-hoc contrasts: week 0 *vs* week 4, p = 0.02, week 4 *vs* week 8, p = 0.01, week 0 *vs* week 8, p = 1.0.

Table S3. Results of general linear model with a Gaussian distribution error to assess whether the change in fat mass or lean mass (% values relative to body mass) between week 0 and week 4 (a-b), or between week 4 and week 8 (c-d) related to the immunological markers measured at week 4 (a-b), or week 8 (c-d). Significant terms (*p* < 0.05) are in bold.

(a) Change in % of fat mass between week 0 and week 4

	Estimate	SE	t	*p*
Intercept	0.101	0.012	8.404	<0.0001
**LCC**	-0.056	0.013	-4.397	0.001
Agglutination	0.001	0.013	0.103	0.919
**Lysis**	-0.066	0.013	-5.202	0.0003

(b) Change in % of lean mass between week 0 and week 4

	Estimate	SE	t	*p*
Intercept	-0.108	0.022	-5.012	<0.0003
**LCC**	0.065	0.023	2.857	0.016
Agglutination	0.009	0.023	0.417	0.685
Lysis	0.040	0.023	1.765	0.105

(c) Change in % of fat mass between week 4 and week 8

	Estimate	SE	t	*p*
Intercept	0.113	0.034	3.299	0.006
LCC	-0.033	0.037	-0.907	0.382
Agglutination	0.065	0.042	1.553	0.146
Lysis	-0.035	0.042	-0.827	0.424

(d) Change in % of lean mass between week 4 and week 8

	Estimate	SE	t	*p*
Intercept	-0.089	0.032	-2.780	0.017
LCC	0.018	0.034	0.538	0.600
Agglutination	-0.078	0.039	-2.017	0.067
Lysis	0.051	0.039	1.305	0.216

Biology Open • Supplementary information
